# Machine Learning-Based Comparative Analysis of Pan-Cancer and Pan-Normal Tissues Identifies Pan-Cancer Tissue-Enriched circRNAs Related to Cancer Mutations as Potential Exosomal Biomarkers

**DOI:** 10.3389/fonc.2021.703461

**Published:** 2021-09-15

**Authors:** Xuezhu Wang, Yucheng Dong, Zilong Wu, Guanqun Wang, Yue Shi, Yongchang Zheng

**Affiliations:** ^1^Department of Liver Surgery, Peking Union Medical College Hospital, Chinese Academy of Medical Sciences and Peking Union Medical College (CAMS & PUMC), Beijing, China; ^2^Peking Union Medical College (PUMC), Chinese Academy of Medical Sciences and Peking Union Medical College (CAMS & PUMC), Beijing, China; ^3^Department of Hepatobiliary Surgery, Hunan Provincial People’s Hospital, The First Affiliated Hospital of Hunan Normal University, Changsha, China; ^4^School of Life Sciences, Tsinghua-Peking Center for Life Sciences, Center for Synthetic and Systems Biology, Ministry of Education Key Laboratory of Bioinformatics, Tsinghua University, Beijing, China

**Keywords:** pan-cancer, circRNA, plasma exosomal biomarkers, machine learning, cancer mutations

## Abstract

A growing body of evidence has shown that circular RNA (circRNA) is a promising exosomal cancer biomarker candidate. However, global circRNA alterations in cancer and the underlying mechanism, essential for identification of ideal circRNA cancer biomarkers, remain under investigation. We comparatively analyzed the circRNA landscape in pan-cancer and pan-normal tissues. Using co-expression and LASSO regularization analyses, as well as a support vector machine, we analyzed 265 pan-cancer and 319 pan-normal tissues in order to identify the circRNAs with the highest ability to distinguish between pan-cancer and pan-normal tissues. We further studied their expression in plasma exosomes from patients with cancer and their relation with cancer mutations and tumor microenvironment landscape. We discovered that circRNA expression was globally reduced in pan-cancer tissues and plasma exosomes from cancer patients than in pan-normal tissues and plasma exosomes from healthy controls. We identified dynein axonemal heavy chain 14 (*DNAH14*), the top back-spliced gene exclusive to pan-cancer tissues, as the host gene of three pan-cancer tissue-enriched circRNAs. Among these three circRNAs, chr1_224952669_224968874_+ was significantly elevated in plasma exosomes from hepatocellular carcinoma and colorectal cancer patients. It was also related to the cancer mutation chr1:224952669: G>A, a splice acceptor variant, and was increasingly transcription-driven in cancer tissues. Moreover, pan-cancer tissue-enriched and pan-normal tissue-enriched circRNAs were associated with distinct tumor microenvironment patterns. Our machine learning-based analysis provides insights into the aberrant landscape and biogenesis of circRNAs in cancer and highlights cancer mutation-related and DNAH14-derived circRNA, chr1_224952669_224968874_+, as a potential cancer biomarker.

## Introduction

Circular RNA (circRNA) is a covalently closed circular and single-stranded non-coding RNA universally generated by cancer and normal cells and has been detected in plasma exosomes derived from these cells (1). CircRNAs are gaining increasing attention as promising cancer biomarkers that can be detected by liquid biopsies and are associated with many cancer types, such as gastric cancer, colorectal cancer (CRC), hepatocellular carcinoma (HCC) and pancreatic adenocarcinoma (PAAD) (2), etc. For example, circ-KIAA1244 was downregulated in gastric tissues and plasma samples in patients with gastric cancer, and this decrease was negatively correlated with the TNM stage, lymphatic metastasis, and overall survival of patients (3). In colon cancer, a scoring model involving four circRNAs effectively predicted the postoperative recurrence of stage II/III cancer (4). Zhang et al. showed that the elevation of circUHRF1 in HCC tissues and plasma exosomes was correlated with poor prognosis and resistance to anti-PD1 immunotherapy (5).

In recent years, studies have revealed great variability of circRNA profiles in pan-cancer and pan-normal tissues, for which numerous circRNA databases have been established (6). The Cancer-Specific CircRNA Database (CSCD) contains circRNA classifications that are “cancer-specific”, “normal-specific” or “common” based on the analysis of hundreds of pan-cancer and pan-normal tissue samples (7). The MiOncoCirc database collects thousands of circRNA profiles in pan-cancer tissues by performing exome capture RNA sequencing (8). The circAtlas database contains circRNA profiles from thousands of samples across 19 different pan-normal tissues, showing that circRNAs can be cell-type specific and species-conserved (9, 10). The exoRbase database is a collection of exosomal circRNA, lncRNA, and mRNA profiles from patients with cancer and healthy controls (11).

To date, the mechanisms underlying circRNA biogenesis remain unclear, particularly those governing aberrant circRNA expression in pan-cancer tissues. Previous findings have supported the back-splicing model, in which the double ends of a pre-mRNA fragment ligate to form a closed circular structure (12), although the driving force and machinery mediating back-splicing remain unclear. The alternative splicing factor Quaking has been implicated in circRNA regulation, as it has been reported to alter circRNA expression during the epithelial-mesenchymal transition, a critical process in cancer metastasis (13). CircRNA formation is also likely associated with H3K79me2 histone modifications (14) that have been shown to regulate co-transcriptional alternative splicing (15).

Back-spliced genes, also called host genes, are often involved in the correlation analysis with circRNA to investigate transcription and back-splicing. The ratio between circRNA level and host gene expression is defined as the junction ratio which is used to evaluate the back-splicing activity (10). The correlations between circRNA and host gene expression were largely positive for the oncogenes of prostate cancer (8). A negative correlation has been observed between circSMARCA5 and SWI/SNF-related matrix-associated actin-dependent regulator of chromatin subfamily A member (*SMARCA5*) in breast cancer tissues and breast cancer cell lines, which indicated the transcriptional pausing of *SMARCA5* induced by circSMARCA5 (16).

Despite the growing body of data in circRNA research, the dysregulations of circRNAs in cancer and the principle of back-splicing remain elusive. Most studies on circRNAs in cancer have not addressed these issues but rather focused on a specific cancer type and para-cancer tissues. They failed to study circRNAs from a pan-cancer view, although the circRNAs related to the common dysregulations in oncogenesis may reveal the principles of circRNA dysregulations in cancer and have higher robustness as therapeutic targets and cancer biomarkers (17). It was ignored that circRNAs are expressed by diverse normal tissues *in vivo* and secreted into plasma exosomes. These constitute the pan-normal tissue-enriched circRNAs which should be excluded from exosomal biomarker candidates.

**Figure 1:**
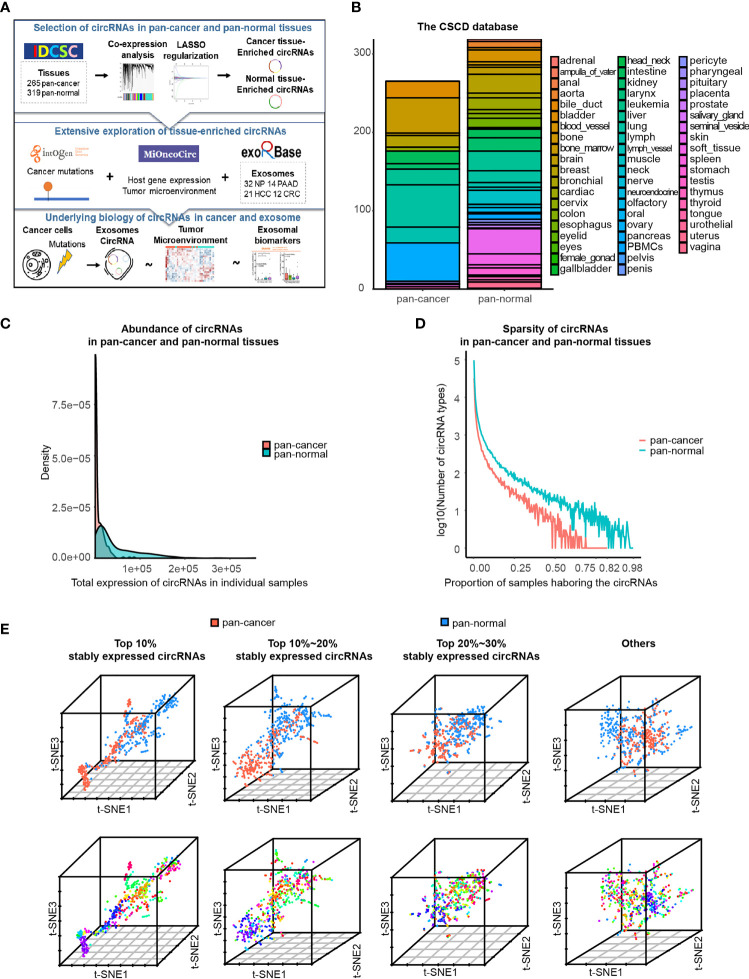
Characteristics of circRNA landscape in pan-cancer and pan-normal tissues provided by CSCD database. **(A)** Figure abstract and flow chart of this study.**(B)** Pan-cancer and pan-normal samples provided by the CSCD database, and classified by cancer types and tissue types. **(C)** Abundance of circRNAs in each pan-cancer and pan-normal tissue sample. **(D)**. Sparsity of circRNA profiles in pan-cancer and pan-normal tissue samples. **(E)** t-SNE embedding of circRNA profiles in 584 tissue samples, after principal component analysis. The top 10%, top 10%–20%, and top 20%–30% of stably expressed circRNAs, and the others were analyzed respectively. Red: pan-cancer tissues; blue: pan-normal tissues. Rainbow: tissue types.

To this end, we performed a comparative analysis to determine the circRNA landscape in pan-cancer and pan-normal tissues and identified pan-cancer tissue-enriched and pan-normal tissue-enriched circRNAs. We examined the expression of pan-cancer tissue-enriched and pan-normal tissue-enriched circRNAs in plasma exosomes from patients with cancer (HCC, CRC, PAAD) and healthy controls. We also studied the relation between circRNAs and cancer mutations, host gene transcription, and tumor microenvironment landscape. Following the conceptual biological process of circRNA biogenesis and secretion, our study successfully integrated the big data of public cancer circRNA profiles (**Figure 1A**).

## Methods

### CSCD Pan-Cancer and Pan-Normal circRNA Profiles

We downloaded circRNA datasets from the highly cited CSCD database (7) (http://gb.whu.edu.cn/CSCD/#). We reorganized the original data format of cancer-specific, normal-specific, and common circRNA counts into the circRNA profiles of individual samples. We selected the circRNA profiles predicted by the CIRCexplorer (18) circRNA prediction algorithm against the GRch38 human reference genome. We removed circRNAs of counts <2 and samples harboring total circRNA counts <10. After removing samples with ambiguous information regarding tissue types, 265 pan-cancer and 319 pan-normal tissues were included (**Additional file Table S1**).

The circRNA nomenclature describes the chromosome, two back-splicing sites, and strandness, such as “chr1_224952669_224968874_+.”

### ExoRbase: Plasma Exosome circRNA Datasets

We downloaded the raw RNA sequencing data of plasma exosomes collected by exoRbase (11) (http://www.exorbase.org/) from the Sequence Read Archive database (https://www.ncbi.nlm.nih.gov/sra/). All HCC, PAAD, CRC patients, and healthy controls were included. We used the CIRCexplorer (18) circRNA prediction algorithm and GRch38 human reference genome to analyze the raw RNA sequencing data for circRNA profiles, removing any circRNAs of counts <2.

### MiOncoCirc: Pan-Cancer and Pan-Normal Gene Expression and circRNA Profiles

We downloaded circRNA datasets from the MiOncoCirc database (8) (https://mioncocirc.github.io/). We reorganized the original data format into the circRNA profiles of individual samples. The circRNA profiles in the MiOncoCirc database were predicted by the CIRCexplorer (18) circRNA prediction algorithm against the GRch38 human reference genome. In total, the circRNA profiles of 876 pan-cancer and 74 pan-normal tissues were included (**Additional file Table S3**). Among these samples, the gene expression profile of 325 pan-cancer and 20 pan-normal tissues were also provided.

We included the following cancer types: multiple myeloma (MM), colon adenocarcinoma (COAD) prostate adenocarcinoma (PRAD), bladder urothelial carcinoma (BLCA), breast invasive carcinoma (BRCA), skin cutaneous melanoma (SKCM), pancreatic adenocarcinoma (PAAD), kidney renal clear cell carcinoma (KIRC), esophageal carcinoma (ESCA), lung squamous cell carcinoma (LUSC), liver hepatocellular carcinoma (LIHC), ovarian serous cystadenocarcinoma (OV), and thyroid carcinoma (THCA). We also included the normal controls (NORM).

### Gene Functional Enrichment

Metascape (19) (http://metascape.org/) is an online tool useful for functional enrichment analysis. We chose the Gene Prioritization by Evidence Counting algorithm and selected the Reactome and Gene Ontology databases. The parameters for pathway and process enrichment were defined as follows: min overlap = 3, p-value (accumulative hypergeometric p-values) cutoff = 0.01, and min enrichments = 1.5.

### LASSO Regularization Analysis

We used the R package “glmnet” (20) to perform the least absolute shrinkage and selection operator (LASSO) regularization analysis, which is a type of machine learning model. For the training set, we randomly selected 70% of pan-cancer and pan-normal tissue samples, with the other 30% comprising a validation set. For LASSO regularization analysis, 50% of the training set was randomly sampled, and LASSO regression was applied for 50 repetitions. Five-fold cross-validation and Akaike information criterion (AIC) analyses were performed to estimate the expected generalization error and the selected optimal value of the “1-se” lambda parameter. An adaptive general linear model to select pan-normal tissue-enriched circRNAs was constructed, with the random seeds being set to 42 to ensure the reproducibility of the results.

### Weighted Gene Co-Expression Network Analysis

We used the R package “WGCNA” (21) to perform the co-expression analysis of circRNAs and identify circRNA co-expression modules that were positively correlated with pan-cancer tissues. The parameters were selected as follows (a): For the top 10% of stably expressed circRNAs: power = 4, max block size = 21,249, min module size = 5, reassign threshold = 0, merge cut height = 0.25, type of correlation = Pearson (b); For the top 10%–20% of stably expressed circRNAs: power = 4, max block size = 23,709, min module size = 5, reassign threshold = 0, merge cut height = 0.25, type of correlation = Pearson (c); For the top 20%–30% of stably expressed circRNAs: power = 6, max block size = 20,768, min module size = 5, reassign threshold = 0, merge cut height = 0.25, type of correlation = Pearson.

### Support Vector Machine

We used the R package “caret” and “e1071” (20) to construct a support vector machine, which is a type of machine learning model. For the training set, we randomly selected 70% of pan-cancer and pan-normal tissue samples, with the other 30% comprising a validation set. We used the training set to train a support vector machine model to perform the binary classification of pan-cancer and pan-normal tissues, and we used the validation set (which was not used for feature selection in the LASSO regularization analysis or support vector machine training) to evaluate the predictive performance of the model. During model training, the performance was improved using the support vector machine tuning function which optimally determined the “gamma” and “cost” parameters by five-fold cross-validation. The performance was then evaluated quantitatively and represented by a receiver operating characteristics curve, which reflected the accuracy of the circRNAs involved in the model to classify pan-cancer and pan-normal tissues. The random seeds were set to 42 to ensure the reproducibility of the results.

### IntOGen: Cancer Mutation Database

IntOGen (22) (https://intogen.org/) database is a compendium of mutational cancer drivers. We used IntOGen to search for potential cancer-associated mutations at the two splice sites of cancer-specific, pan-cancer tissue-enriched, and pan-normal tissue-enriched circRNAs. The human reference genome used for this analysis was GRch38.

The cancer mutation nomenclature describes the genomic position and nucleotide variant, such as “chr1:224952669:G>A”.

### xCell: Cell Types Enrichment Analysis

xCell (23) (https://xcell.ucsf.edu/) is a method learned from thousands of pure cell types from various sources, which performs cell type enrichment analysis based on gene expression in 64 immune and stromal cell types. We used xCell to infer the abundance of 64 cell types from the gene expression profile of cancer and normal tissues provided by MiOncoCirc.

### Statistical Analysis

We used R software (Version 3.6.0) algorithms to conduct basic visualization and statistical analysis, including density, violin, bar, and line plots, Venn diagrams, heatmaps, t-distributed stochastic neighbor embedding (t-SNE), and principal component analysis (PCA). The Session Info of R software can be found in the **Supplementary Material** and the GitHub repository.

## Results

### CircRNAs Are Less Abundant and Less Stably Expressed in Pan-Cancer Tissues Compared With the Pan-Normal Tissues

In total, we used 265 pan-cancer tissue samples across 15 different tissue types and 319 pan-normal tissue samples from 38 anatomical sites, provided by the CSCD database (7) (**Figure 1B** and **Additional File Table S1**). The abundance of circRNAs in pan-cancer tissues was significantly lower than that in the pan-normal tissues, and the latter showed a greater range of expression (**Figure 1C**). Most pan-cancer tissues harbored extremely low circRNA levels, whereas some pan-normal tissue expressed very high circRNA levels. The number of circRNA types did not increase with the increase in the total counts of circRNAs (**Additional File Figure S1A**), suggesting that the nature of tumorigenesis, rather than the sequencing depth, was the underlying cause.

Most circRNAs were expressed at low levels in the analyzed tissues. Of the combined samples (584 in total), the top 10% of stably expressed circRNAs occurred in ≥20 samples, top 20% in ≥7 samples, top 30% in ≥4 samples, and top 40% in ≥2 samples. Approximately 50% of circRNAs occurred in only one of the 584 samples. This sparsity of circRNA expression was more prominent in pan-cancer tissues than in pan-normal tissues (**Figure 1D**).

Based on the hypothesis that more commonly expressed circRNAs have a higher potential to serve as biomarkers, a total of 210,784 circRNAs were divided into four groups: the top 10%, top 10%–20%, and top 20%–30% stably expressed and other less stably expressed circRNAs. t-SNE embedding of the four groups of top stably expressed circRNA profiles demonstrated that samples from the same tissue type tended to be neighbors. t-SNE embedding of the top 10% stably expressed circRNAs showed the most distinct separation of the different sample types, regardless of whether PCA was performed (**Figure 1E** and **Additional File Figure S1B**). These results support previous observations that circRNA expression exhibits high tissue type-specificity (10). Therefore, downstream analyses were employed separately for the different expression groups (top 10%, 10%–20%, and 20%–30% of stably expressed circRNAs).

### Pan-Cancer and Pan-Normal Tissues Share a Large Proportion of Top Actively Back-Spliced Genes and Show Differences in Functional Enrichment

**Figure 2:**
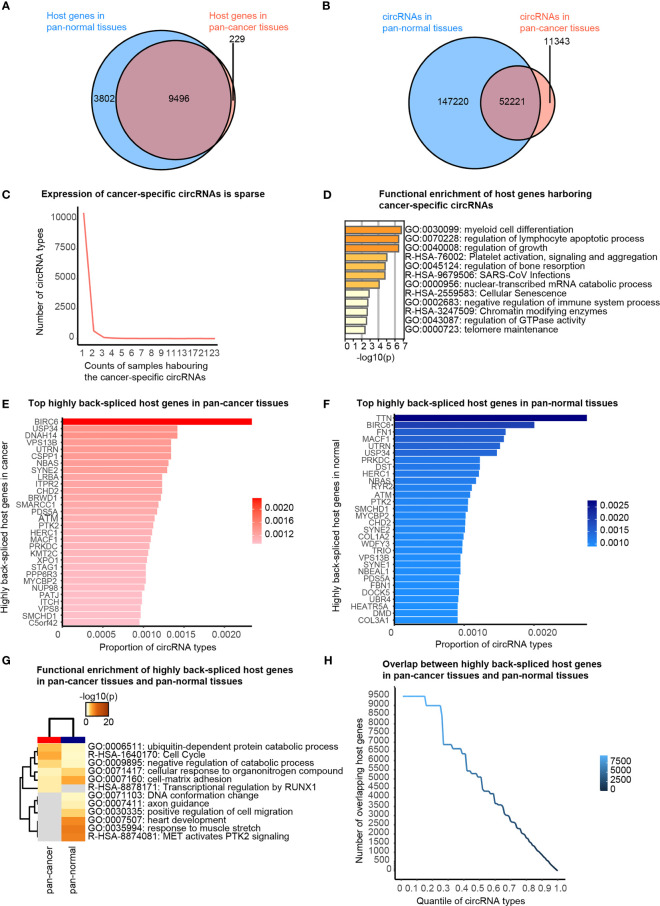
Cancer-specific circRNAs and host genes, and top actively back-spliced host genes. **(A)** Venn diagram showing the overlap between the host genes of circRNAs in pan-cancer and pan-normal tissues. **(B)** Venn diagram showing the overlap between circRNA profiles of pan-cancer and pan-normal tissues. **(C)** Cancer-specific circRNAs harbored by different counts of samples. **(D)** Functional enrichment of the host genes of the stably expressed cancer-specific circRNAs. **(E)** Top 30 actively back-spliced host genes in pan-cancer tissues. Proportion of total circRNA types related to the same host gene represents the back-splicing activity. **(F)** Top 30 actively back-spliced host genes in pan-normal tissues. **(G)** Functional enrichment of the top 30 actively back-spliced host genes in the pan-cancer and pan-normal tissues. **(H)** Overlap between the top actively back-spliced host genes in pan-cancer and pan-normal tissues.

Firstly, we briefly revisited the concept of cancer-specific circRNAs, which indicates the circRNAs observed exclusively in pan-cancer tissues, as defined by the CSCD database. About 97.65% of circRNA host genes observed in the pan-cancer tissues were also detected in pan-normal tissues (**Figure 2A**). A total of 82.16% circRNAs present in pan-cancer tissues were also observed in pan-normal tissues (**Figure 2B**). Most of the 11,343 cancer-specific circRNAs were not stably expressed, and only 74 circRNAs were stably expressed in ≥4 tissues (**Figure 2C** and **Additional file Table S2**). Interestingly, the host genes of these 74 circRNAs displayed functional enrichment in myeloid cell differentiation, regulation of lymphocyte apoptotic process, and growth regulation, which are likely related to oncogenesis (**Figure 2D**).

We also found that some genes were more actively back-spliced, thereby serving as host genes of a greater number of differentially expressed circRNAs. The top 30 actively back-spliced host genes in pan-cancer (**Figure 2E**) and pan-normal tissues showed prominent overlap (**Figure 2F**), despite the ranking difference. Dynein axonemal heavy chain 14 (*DNAH14*) was the third most actively back-spliced gene exclusive in pan-cancer tissues. Titin (*TTN*) was the top actively back-spliced gene exclusive in pan-normal tissues. *TTN* was recently reported to serve as a host gene for regulatory circRNAs with important roles in the splicing of muscle genes in the human heart (24). Although functional enrichment analysis of the top 30 highly spliced host genes showed prominent overlap, those in pan-cancer tissues were enriched in the ubiquitin-dependent protein catabolic process, cell cycle, and negative regulation of the catabolic process. In contrast, those in pan-normal tissues were enriched in “MET activates PTK2 signaling”, “response to muscle stretch”, “heart development”, “cell-matrix adhesion”, and “cellular response to organonitrogen compounds” (**Figure 2G**). The overlap between the top actively back-spliced host genes in pan-cancer and pan-normal tissues increased steadily as the ranking quantile increased, whereas the least actively back-spliced host genes (ranking quantile <0.3) also showed significantly increased overlap (**Figure 2H**).

### Pan-Normal Tissue-Enriched circRNAs Are Associated With Universal Cellular Functions

Given that cancer-specific circRNAs were not likely a good cancer biomarker, we aimed to screen for circRNAs with the highest ability to distinguish the pan-cancer and pan-normal tissues, which were the candidates for plasma exosomal cancer biomarker.

**Figure 3:**
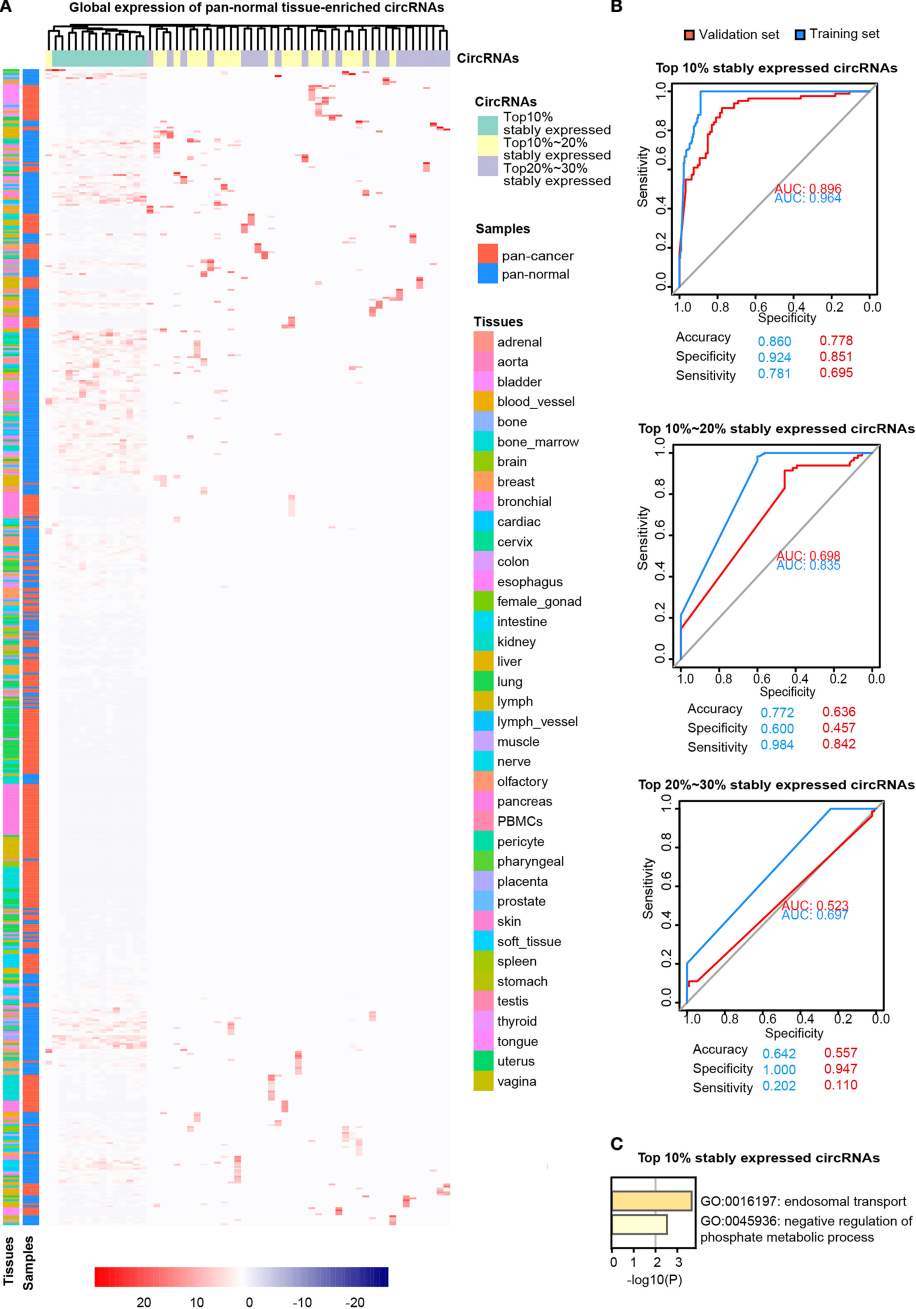
Pan-normal tissue-enriched circRNAs. **(A)** Heatmap illustrating the expression of pan-normal tissue-enriched circRNAs selected by LASSO regularization analysis among the top 10%, top 10%–20%, and top 20%–30% stably expressed circRNAs. Turquoise: top 10%; yellow: top 10%–20%; gray: top 20%–30%. Red: pan-cancer tissues; blue: pan-normal tissues; rainbow: tissue types. **(B)** Receiver operating characteristic curve showing the sensitivity of pan-normal tissue-enriched circRNAs to classify pan-cancer and pan-normal tissues *via* the support vector machine. The top 10%, top 10%–20%, and top 20%–30% of stably expressed circRNAs were separately analyzed. Blue: training set; red: validation set. **(C)** Functional enrichment of the host genes of pan-normal tissue-enriched circRNAs among the top 10% of stably expressed circRNAs.

Pan-normal tissue-enriched circRNAs were selected by LASSO regularization analysis, from the top 10%, 10%–20%, and 20%–30% stably expressed circRNAs (**Additional files Figures S2A, B**). We selected the 14 pan-normal tissue-enriched circRNAs among the top 10% of stably expressed circRNAs (**Figure 3A**) that exhibited the strongest ability to classify pan-cancer and pan-normal tissues (**Figure 3B**). The pan-normal enriched circRNAs were universally and stably expressed in the pan-normal tissues, while they were almost not observed in the pan-cancer tissues. These pan-normal tissue-enriched circRNAs were derived from protein-coding host genes (**Table 1**), which were enriched in universal cellular functions, including endosomal transport and the phosphate metabolic process (**Figure 3C**).

**Table 1:** Pan-normal tissue-enriched circRNAs derived from protein-coding genes.

circRNA	Host gene name (Protein-coding)
chr1_155438326_155459898_-	ASH1L
chr1_1804418_1817875_-	GNB1
chr10_1072115_1096246_+	WDR37
chr11_36227084_36227430_+	LDLRAD3
chr12_123498543_123499536_-	RILPL1
chr14_24266429_24268619_-	RABGGTA
chr16_11020192_11126146_+	CLEC16A
chr17_31940285_31940486_+	SUZ12
chr3_114350273_114351878_-	ZBTB20
chr3_172247532_172251541_+	FNDC3B
chr4_40934455_40945070_-	APBB2
chr5_38523418_38530666_-	LIFR
chr6_138943512_138944622_-	REPS1
chr7_98190727_98194572_+	LMTK2

### Pan-Cancer Tissue-Enriched circRNAs Are Predominantly Back-Spliced From Oncogenes

**Figure 4:**
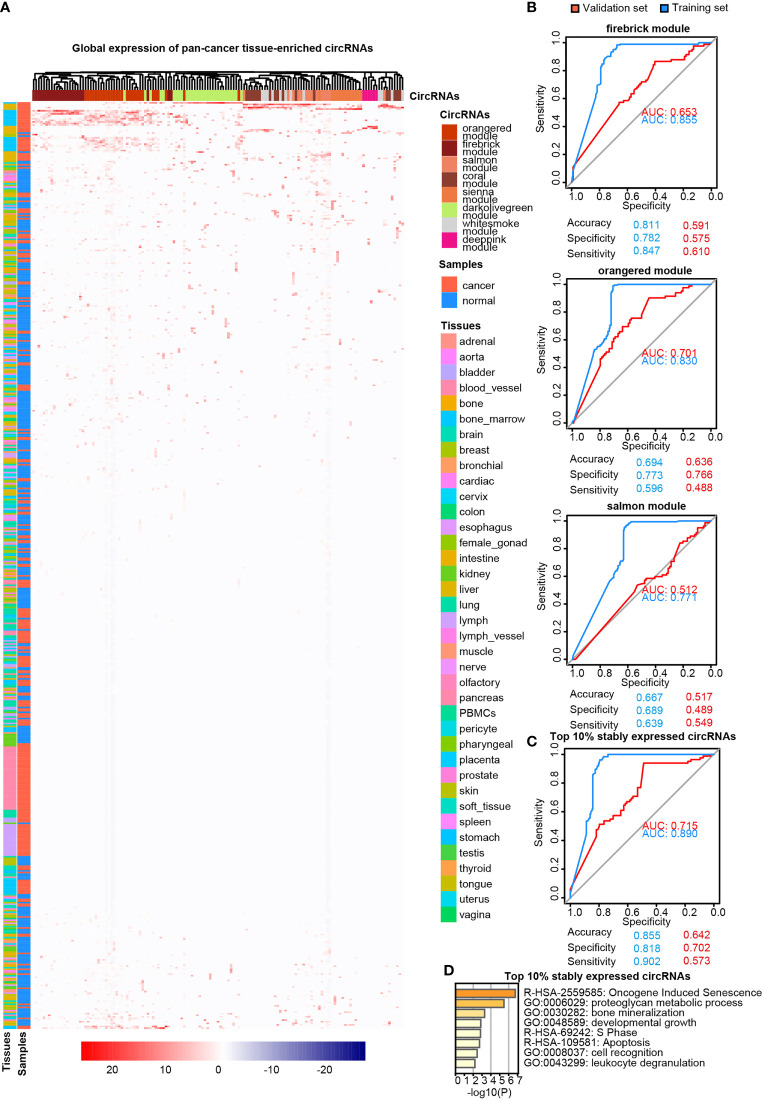
Pan-cancer tissue-enriched circRNAs. **(A)** Heatmap illustrating the expression of pan-cancer tissue-enriched circRNAs identified by co-expression analysis. Firebrick, orange-red, salmon, white-smoke, sienna, dark-olive-green, coral, and deep-pink represent co-expression modules. Red: pan-cancer tissues; blue: pan-normal tissues; rainbow: tissue types. **(B)** Receiver operating characteristic curve showing the sensitivity of pan-cancer tissue-enriched circRNAs to classify pan-cancer and pan-normal tissues *via* the support vector machine. Different circRNA co-expression modules were analyzed. Blue: training set; red: validation set. **(C)** Receiver operating characteristic curve showing the sensitivity of pan-cancer tissue-enriched circRNAs to classify pan-cancer and pan-normal tissues *via* the support vector machine. These pan-cancer tissue-enriched circRNAs were selected from the firebrick, orange-red, and salmon co-expression modules. Blue: training set; red: validation set. **(D)** Functional enrichment of the host genes of pan-cancer tissue-enriched circRNAs among the top 10% stably expressed circRNAs, which are the firebrick, orange-red, and salmon co-expression modules.

LASSO regularization analysis was not adequate to identify pan-cancer tissue-enriched circRNAs potentially due to their low abundance and sparsity. Therefore, we performed co-expression analysis to identify pan-cancer tissue-enriched circRNAs. Co-expression modules positively correlated with cancer were the firebrick, orange-red, and salmon modules in the top 10% of stably expressed circRNA group; white-smoke, sienna, and dark-olive-green modules in the top 10%–20% of stably expressed circRNA group; and coral and deep-pink modules in the top 20%–30% of stably expressed circRNA group (**Additional Files Figures S3A, B**). The enrichment of these pan-cancer tissue-enriched circRNAs in cancer tissues was observed, although with variations between different cancer types. They were most stably elevated in HCC and T-cell acute lymphoblastic leukemia bone marrow, whereas they were unstably expressed in pancreatic and kidney cancers (**Figure 4A**). Similar to pan-normal tissue-enriched circRNAs, pan-cancer tissue-enriched circRNAs selected from the top 10% stably expressed circRNAs showed the strongest ability to distinguish pan-cancer tissues from pan-normal tissues (**Figure 4B** and **Additional File Figure S4A**). The 22 pan-cancer tissues-enriched circRNAs from the top 10% stably expressed circRNAs were further selected by LASSO regularization analysis (**Figure 4C** and **Additional Files Figures S4B, C**), among which 18 circRNAs were most pan-cancer tissues-enriched and were derived from protein-coding host genes (**Table 2**). Interestingly, the host genes of pan-cancer tissue-enriched circRNAs and top 10% stably expressed circRNAs were most significantly enriched in “oncogene-induced senescence” which implicated the tendency of oncogenes to be circRNA host genes in cancer tissues (**Figure 4D**).

**Table 2:** Pan-cancer tissue-enriched circRNAs derived from protein-coding genes.

circRNA	Host gene name (Protein-coding)
chr1_224952669_224968874_+	DNAH14
chr1_224952669_224974153_+	DNAH14
chr1_225231072_225266769_+	DNAH14
chr10_110964124_110965061_+	SHOC2
chr10_42631928_42636972_-	ZNF33B
chr2_45546731_45553730_-	SRBD1
chr2_55025085_55028220_-	RTN4
chr20_13483226_13587370_-	TASP1
chr20_13559007_13580981_-	TASP1
chr20_13559007_13587370_-	TASP1
chr20_13569506_13587370_-	TASP1
chr4_118105017_118114960_+	NDST3
chr4_177353307_177360677_+	NEIL3
chr5_109781395_109789527_+	MAN2A1
chr5_36207169_36227565_-	NADK2
chr7_116110707_116112038_-	TFEC
chr8_96879797_96880005_+	CPQ
chrX_6150994_6151771_-	NLGN4X

### Increased Levels of Pan-Cancer Tissue-Enriched circRNAs Related to Cancer Mutations Are Present in Plasma Exosomes From Patients With Cancer

**Figure 5:**
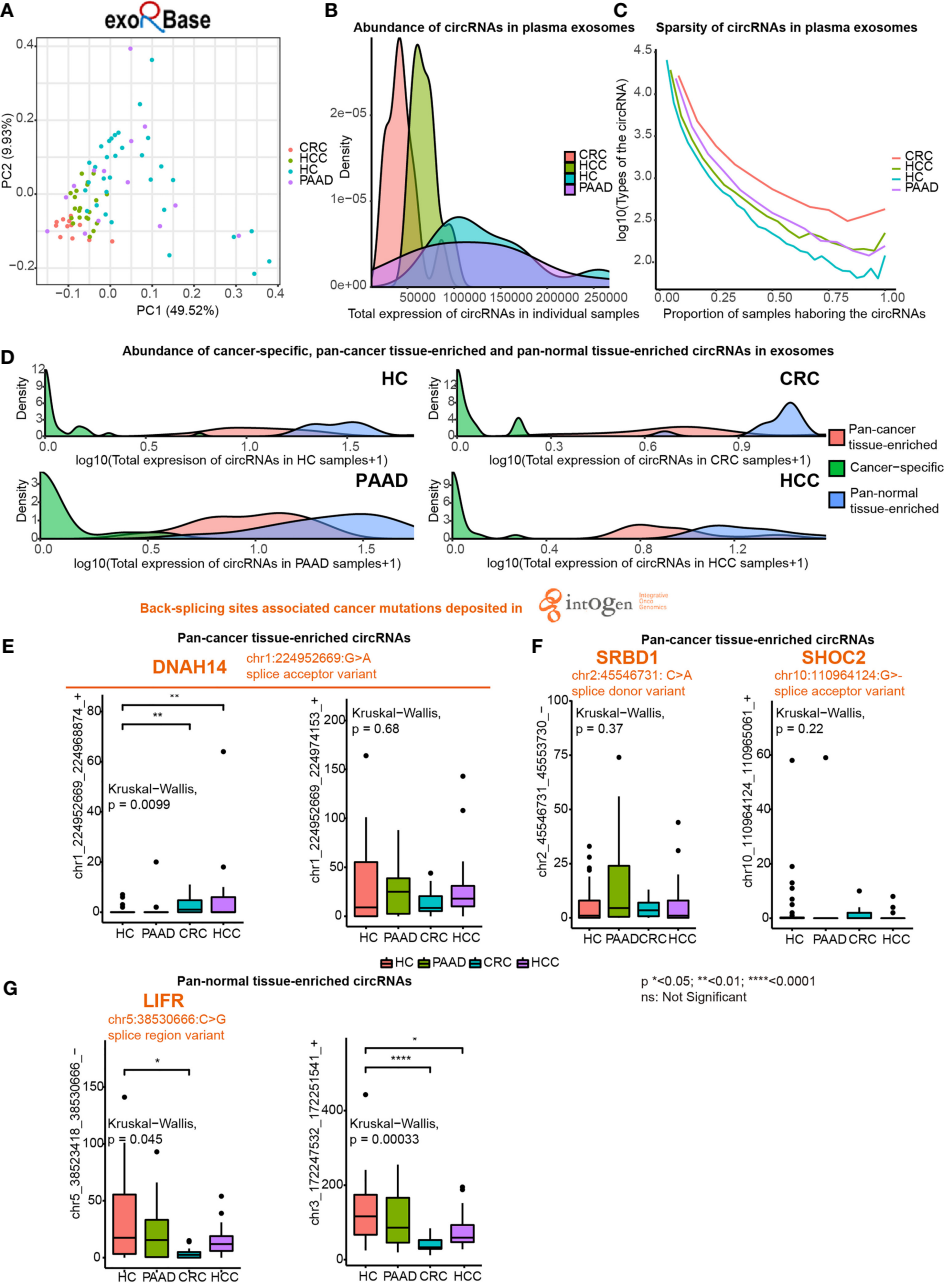
Expression of pan-cancer tissue-enriched and pan-normal tissue-enriched circRNAs in plasma exosomes based on exoRbase data. **(A)** Principle component analysis of plasma exosomal circRNA profiles of healthy controls (HC), hepatocellular carcinoma patients (HCC), colorectal cancer patients (CRC), and pancreatic adenocarcinoma patients (PAAD). **(B)** Abundance of circRNAs in plasma exosomes from HC, HCC, CRC, and PAAD. **(C)** Sparsity of circRNA profiles in plasma exosomes from HC, HCC, CRC, and PAAD. **(D)** Abundance of cancer tissue-specific circRNAs, pan-cancer tissue-enriched circRNAs, and pan-normal tissue-enriched circRNAs in HC, HCC, CRC, and PAAD. **(E)** Expression of chr1:224952669:G>A (*DNAH14*)-related circRNAs in plasma exosomes from HC, HCC, CRC, and PAAD. **(F)** Expression of other cancer mutation-related pan-cancer tissue-enriched circRNAs in plasma exosomes from HC, HCC, CRC, and PAAD. **(G)** Expression of pan-normal tissue-enriched circRNAs in plasma exosomes from HC, HCC, CRC, and PAAD.

Next, we investigated pan-cancer tissue-enriched and pan-normal tissue-enriched circRNAs in plasma exosomes by analyzing all the healthy control (HC), pancreatic adenocarcinoma (PAAD), colorectal cancer (CRC), and hepatocellular carcinoma (HCC) datasets collected by the exoRBase database. The diversity of circRNA profiles in plasma exosomes from CRC and HCC patients was lower than those from PAAD and NP (**Figure 5A**). Furthermore, circRNAs were less abundant in the plasma exosome from CRC and HCC patients (**Figure 5B**). The sparsity of circRNA profiles was also observed in plasma exosomes but was different from the higher sparsity of circRNA expression in pan-cancer tissues (**Figure 1D**); the circRNA profiles in plasma exosomes from cancer patients were more stable (**Figure 5C**). These differences in plasma exosomal circRNA profiles among cancer types have not been previously reported.

We found that the abundance of cancer-specific, pan-cancer tissue-enriched and pan-normal tissue-enriched circRNAs was different in plasma exosomes. Pan-normal tissue-enriched circRNAs were the most abundant, while cancer-specific circRNAs were the least abundant, and pan-cancer tissue-enriched circRNAs were intermediate (**Figure 5D**). This observation supported that cancer-specific circRNAs were not a good candidate for plasma exosomes and that pan-normal tissue-enriched circRNAs were universally expressed and secreted by normal tissues.

Thereafter, we studied the differential expression of pan-cancer tissue-enriched and pan-normal tissue-enriched circRNAs in the plasma exosomes from healthy controls and patients with PAAD, CRC, and HCC. Specifically, the circRNA chr1_224952669_224968874_+ related to a splice acceptor variant of *DNAH14* (chr1:224952669:G>A) was a pan-cancer tissue-enriched circRNA significantly elevated in plasma exosomes from CRC and HCC patients. Related to the same cancer mutation, the circRNA chr1_224952669_224974153_+ was highly expressed in the plasma exosomes of both healthy controls and cancer patients (**Figure 5E**). chr2:45546731: C>A was a splice donor variant of the S1 RNA binding domain 1 (*SRBD1*), and the circRNA chr2_45546731_45553730_- was highly expressed in the plasma exosomes from PAAD. chr10_110964124_110965061_+ was elevated in the plasma exosomes from CRC, related to chr10:110964124:G>- that was a splice acceptor variant of SHOC2 leucine-rich repeat scaffold protein (*SHOC2*) (**Figure 5F**). In contrast, no splice donor variants or splice acceptor variants were related to pan-normal tissue-enriched circRNAs, despite a splice region variant of LIF receptor subunit alpha (*LIFR*) (chr5:38530666:C>G) being related to the pan-normal tissue-enriched chr5:38523418_38530666_- (**Figure 5G**).

### Expression of Pan-Cancer Tissue-Enriched circRNAs Is Increasingly Transcription-Driven in Cancer Tissues and Correlated With Tumor Microenvironment Landscape

**Figure 6:**
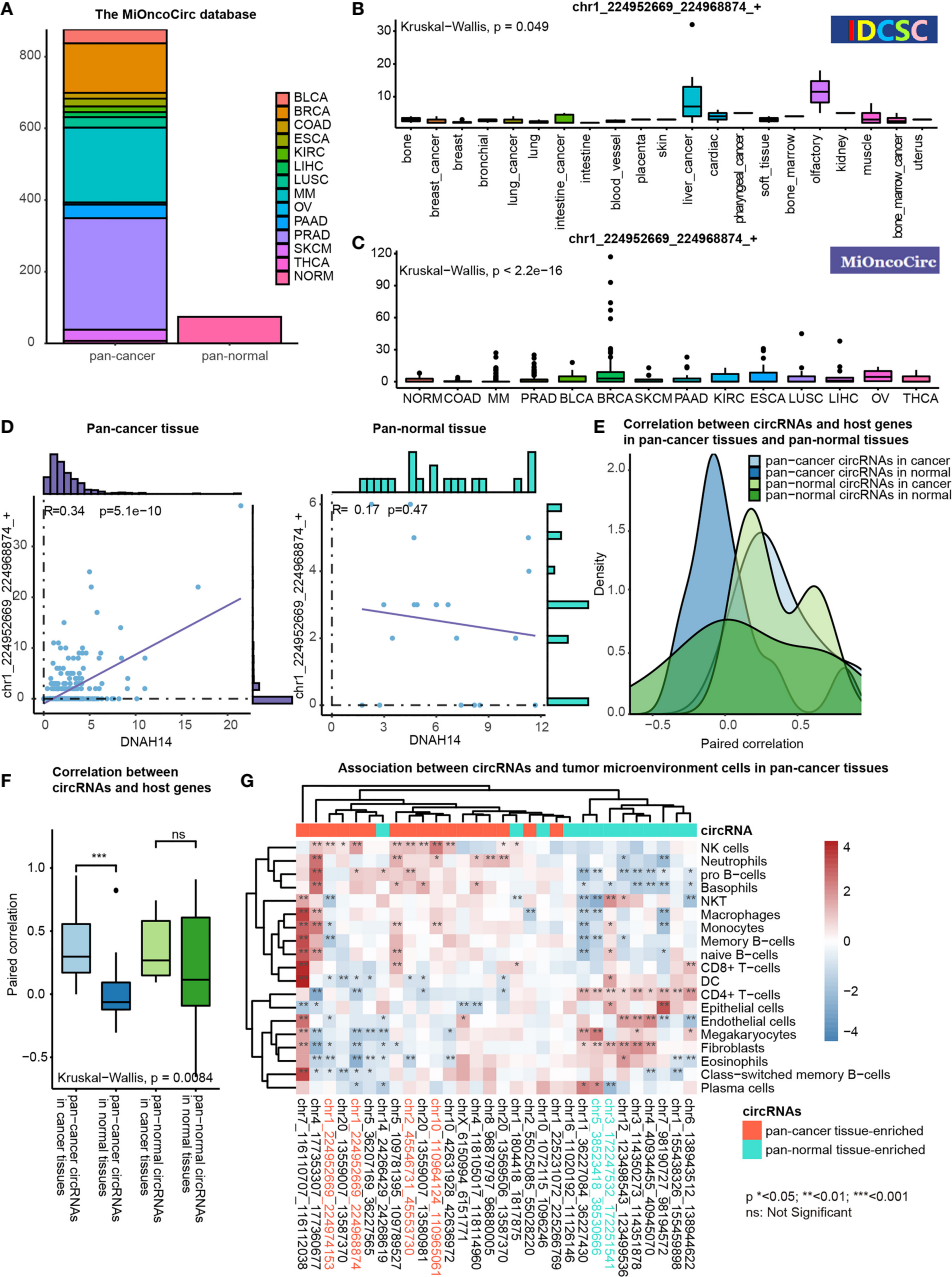
Functional association studies of pan-cancer tissue-enriched circRNAs using MiOncoCirc data. **(A)** Pan-cancer and pan-normal samples provided by the MiOncoCirc database including multiple myeloma (MM), colon adenocarcinoma (COAD) prostate adenocarcinoma (PRAD), bladder urothelial carcinoma (BLCA), breast invasive carcinoma (BRCA), skin cutaneous melanoma (SKCM), pancreatic adenocarcinoma (PAAD), kidney renal clear cell carcinoma (KIRC), esophageal carcinoma (ESCA), lung squamous cell carcinoma (LUSC), liver hepatocellular carcinoma (LIHC), ovarian serous cystadenocarcinoma (OV) and thyroid carcinoma (THCA), together with normal controls (NORM).d **(B)** Expression of chr1_224952669_224968874_+ in the samples collected by the CSCD database. **(C)** Expression of chr1_224952669_224968874_+ in the samples collected by the MiOncoCirc database. **(D)** Correlation between chr1_224952669_224968874_+ level and *DNAH14* expression in pan-cancer and pan-normal tissues. **(E)** Distribution of paired correlation scores between pan-cancer tissue-enriched or pan-normal tissue-enriched circRNAs and host genes in pan-cancer tissues and pan-normal tissues; **(F)** Alteration of paired correlation scores between pan-cancer tissue-enriched or pan-normal tissue-enriched circRNAs and host genes during oncogenesis; **(G)** Correlation between pan-cancer tissue-enriched circRNAs and the abundance of tumor microenvironment cells in pan-cancer tissues. Red: high Spearman correlation score; blue: low Spearman correlation score.

We characterized the potential functions of pan-cancer tissue-enriched circRNA chr1_224952669_224968874_+ by analyzing the circRNA profiles and gene expression profiles provided by the MiOncoCirc database (8). Altogether, 879 pan-cancer and 77 pan-normal tissues from the MiOncoCirc database were included (**Figure 6A**). We observed that chr1_224952669_224968874_+ was elevated in most types of pan-cancer tissues in the CSCD and MiOncoCirc database (**Figures 6B, C**).

chr1_224952669_224968874_+ was more transcription-driven in the pan-cancer tissue than in the pan-normal tissues. chr1_224952669_224968874_+ was 2- to 4-fold elevated in pan-cancer tissues, while the expression level of *DNAH14* was similar in pan-cancer and pan-normal tissues. Interestingly, chr1_224952669_224968874_+ levels were positively correlated with *DNAH14* expression in the pan-cancer tissues but not in the pan-normal tissues (**Figure 6D**).

This altered host gene correlation was observed in the other pan-cancer tissue-enriched circRNAs but not in pan-normal tissue-enriched circRNAs. The correlation between pan-cancer tissue-enriched circRNAs and their host genes was significantly higher in the cancer tissue than in the normal tissues but this change was not observed among pan-normal tissue-enriched circRNAs. (**Figures 6E, F**). The average correlation was positive in cancer tissues, but negative in normal tissues. These data revealed that pan-cancer tissue-enriched circRNAs were increasingly transcription-driven in pan-cancer tissues, the underlying biology of which was potentially the cancer mutations near the circRNA splicing sites.

Moreover, pan-cancer tissue-enriched and pan-normal tissue-enriched circRNAs were associated with distinct tumor microenvironment patterns. The pan-cancer tissues highly expressing pan-cancer tissue-enriched circRNAs tended to recruit a greater abundance of NK cells, neutrophils, pro-B cells, etc. The expression of pan-normal tissue-enriched circRNAs was positively correlated with a different group of tumor microenvironment cells, including the CD4+ T-cells, endothelial cells, and fibroblasts. This result indicated that the expression level of pan-cancer tissue-enriched and pan-normal tissue-enriched circRNAs was indicative of different tumor microenvironment patterns (**Figure 6G**).

## Discussion

To the best of our knowledge, there has been no machine learning-based comparative analysis of circRNAs in pan-cancer and pan-normal tissues or reports regarding the potential relationship between cancer mutations and circRNAs. Herein, we identified pan-cancer tissue-enriched and pan-normal tissue-enriched circRNAs and studied their expression in plasma exosomes, associated with host gene expression and tumor microenvironment landscape, to account for the fact that circRNAs in plasma exosomes are secreted by a wide variety of pan-normal and pan-cancer tissues (**Figure 1A**). We chose the CSCD database in the machine learning-based analysis because it contained a relatively balanced number of pan-cancer and pan-normal tissues. We used the datasets in the MiOncoCirc database to validate pan-cancer tissue-enriched circRNAs and used the corresponding gene expression profile for integrative analysis. We used the plasma exosomal RNA sequencing profile from CRC, PAAD, and HCC patients, which were collected from the exoRbase database.

Recently, several studies have investigated circRNAs from a pan-cancer view but implemented different methods. Based on a circRNA-miRNA-mRNA network in pan-cancer, Chen et al. discovered that the overexpression of hsa_circ_0004639 and down-regulation of hsa_circ_0008310 could decrease the malignancy of cancer cells which were supported by experimental evidence (25). Analyses of the pan-cancer dataset from the MiOncoCirc database associated CDR1as with angiogenesis, extracellular matrix organization, integrin binding, and collagen binding, as well as the composition of immune and stromal cells in the tumor microenvironment (26). Different from these studies, we innovatively used machine learning-based methods to screen for pan-cancer tissue-enriched and pan-normal tissue-enriched circRNAs and further investigated their expression in plasma exosomes from cancer patients. Our research is less dependent on prior knowledge compared with previous studies.

First, we revisited the concept of cancer-specific circRNAs (circRNAs expressed in pan-cancer tissues, but not in pan-normal tissues), as proposed by the CSCD database (7). Overall, “cancer-specific” was not an ideal criterion for screening circRNA cancer biomarkers. Most cancer-specific circRNAs were expressed very unstably in pan-cancer tissues (**Figure 2C**) and were at very low levels in plasma exosomes (**Figure 5D**). Pan-cancer tissue-enriched circRNAs were more stably expressed in cancer tissues, and their host genes were enriched in the “oncogene-induced senescence” (**Figure 4D**). Oncogene-induced senescence is a cellular system responsive to oncogenic signaling, which is reported to be a “double-edged sword” that can either induce or inhibit oncogenesis (27). Pan-normal tissue-enriched circRNAs were universally and stably expressed in various pan-normal tissues but rarely expressed in the pan-cancer tissues, suggesting that these circRNAs were lost during tissue transition from normal to cancerous.

The abundance of circRNAs was less in pan-cancer than in pan-normal tissues (**Figure 1C**). The total number of circRNA types did not elevate with increasing total counts of circRNAs, suggesting that the sequencing depth was not the reason for this difference (**Figure S1A**). Since circRNAs are relatively long-lived RNA molecules, the rapid proliferation of cancer cells may lead to a decreased abundance of circRNAs, as observed in colorectal and ovarian cancer (28). Furthermore, the changes in the level of splicing factors involved in circRNA biogenesis may contribute to a decreased level of circRNAs (29). Unlike the tissues, the abundance of circRNAs in plasma exosomes was higher in the healthy controls and patients with PAAD but lower in the patients with CRC and HCC (**Figure 5B**). The possibly related evidence is that pancreatic adenocarcinoma is a tumor with a relatively low blood supply, which hinders the secretion of exosomes harboring circRNAs into the plasma. Consequently, the plasma exosome of patients with PAAD was more like that of healthy controls.

We highlighted the potential role of *DNAH14* as an important host gene of circRNAs in cancer. *DNAH14* was the third-highest back-spliced host gene in pan-cancer tissues but was not among the top back-spliced host genes in the pan-normal tissues, although the overlap between the top back-spliced genes in pan-cancer and pan-normal tissues was prominent (**Figures 2E**, **F**). The pan-cancer tissue-enriched chr1_224952669_224968874_+ and chr1_224952669_224974153_+ were related to the splice acceptor variant of *DNAH14* (chr1:224952669:G>A). Particularly, chr1_224952669_224968874_+ was elevated in pan-cancer tissues compared with the pan-normal tissues, supported by the CSCD and MiOncoCirc databases (**Figures 6B, C**). It was significantly elevated in plasma exosomes from patients with HCC and CRC, which indicated its potential role as a plasma cancer biomarker. Although *DNAH14* was not upregulated in cancer tissues, chr1_224952669_224968874_+ was elevated and increasingly transcription-driven (**Figure 6D**). Here, we hypothesized that the splice acceptor variant chr1:224952669:G>A (*DNAH14*) is relatively frequent in cancer tissues and cancer-specific circRNAs. It is a potential driving force facilitating the biogenesis of circRNA chr1_224952669_224968874_+ in cancer tissues.

*DNAH14* encodes a microtubule-associated motor protein that participates in maintaining the integrity of centrosomes, and it is often numerically, positionally, or structurally dysregulated in cancer (30). Dynein encoding genes (*DNAH* family) are among the most frequently mutated genes in cancer (22). In recent studies, somatic mutations in *DNAH* genes have been associated with a higher chemotherapy response rate in patients with gastric cancer (31). These findings and the literature highlight that *DNAH14* as a host gene should be further examined in researches on circRNAs in cancer.

There were several limitations to our study. First, we did not analyze pan-cancer plasma exosome circRNA profiles, as the resources of RNA sequencing data of plasma exosomes from patients with cancer are limited. Second, the cancer mutations were not inferred from the pan-cancer tissue samples that we used, as genomic mutation data was not provided by the current circRNA databases. In future studies, a collection of circRNA profiles, genomic mutation data, and gene expression profile of cancer tissues, together with the plasma exosome circRNA profiles, in a pan-cancer patient cohort is warranted.

## Conclusion

Our machine learning-based analysis of pan-cancer and pan-normal tissues provides insights into the aberrant landscape and biogenesis of circRNAs in cancer. Our results highlight the cancer mutation-related and DNAH14-derived circRNA, chr1_224952669_224968874_+, as a potential cancer biomarker.

## Data Availability Statement

The original contributions presented in the study are included in the article/**Supplementary Material**. The data and codes are available in the GitHub repository (https://github.com/Selecton98/CircRNA_pan-cancer).

## Author Contributions 

XW conceptualized the study, analyzed the data, and drafted the work. YD and ZW analyzed the data and revised the work. GW and YS collected the data. YZ designed the project and revised the work. All authors contributed to the article and approved the submitted version.

## Funding

This study was supported by grants from Chen Xiao Ping Foundation for the Development of Science and Technology of Hubei Province (CXPJJH1200008-10), WBE Liver Fibrosis Foundation (CFHPC 2020021), Beijing Dongcheng District outstanding talent funding project, the Beijing Undergraduate Training Programs for Innovation and Entrepreneurship (202010023046) and the National Undergraduate Training Programs for Innovation and Entrepreneurship (202110023012).

## Conflict of Interest

The authors declare that the research was conducted in the absence of any commercial or financial relationships that could be construed as a potential conflict of interest.

## Publisher’s Note

All claims expressed in this article are solely those of the authors and do not necessarily represent those of their affiliated organizations, or those of the publisher, the editors and the reviewers. Any product that may be evaluated in this article, or claim that may be made by its manufacturer, is not guaranteed or endorsed by the publisher.
