# Supplementary material for: Machine Learning-Based Comparative Analysis of Pan-Cancer and Pan-Normal Tissues Identifies Pan-Cancer Tissue-Enriched circRNAs Related to Cancer Mutations as Potential Exosomal Biomarkers
Source: Front Oncol. 2021 Sep 15;11:703461. doi: 10.3389/fonc.2021.703461 (PMC8479194; doi:10.3389/fonc.2021.703461)
Supplement: Supplementary file 2 [file DataSheet_1.docx]

Supplementary Material

**Machine Learning-based Comparative Analysis of Pan-cancer and Pan-normal Tissues Identifies Pan-cancer Tissue-enriched circRNAs Related to Cancer Mutations as Potential Exosomal Biomarkers**

Xuezhu Wang^1,2^, Yucheng Dong^1,2^, Zilong Wu^3^, Guanqun Wang^4^, Yue Shi^1,2^, Yongchang Zheng^1*^

^1^Department of Liver Surgery, Peking Union Medical College Hospital, Chinese Academy of Medical Sciences and Peking Union Medical College (CAMS & PUMC), Beijing, 100730, China

^2^Peking Union Medical College (PUMC), Chinese Academy of Medical Sciences and Peking Union Medical College (CAMS & PUMC), Beijing, 100730, China.

^3^Department of Hepatobiliary Surgery, Hunan Provincial People's Hospital, the First Affiliated Hospital of Hunan Normal University, Changsha, 410005, China

^4^School of Life Sciences, Tsinghua-Peking Center for Life Sciences, Center for Synthetic and Systems Biology, Ministry of Education Key Laboratory of Bioinformatics, Tsinghua University, 100084 Beijing, China

***To whom correspondence should be addressed. Email: yong-chang_zheng@outlook.com**

**Table of Contents**

| Fig S1-S4 | Page 2-7 |
| --- | --- |
| Table S1-S4 | Additional File 2 |
| Metrics of the Support Vector Machines | Page 9-16 |
| Session information of R software | Page 17-18 |


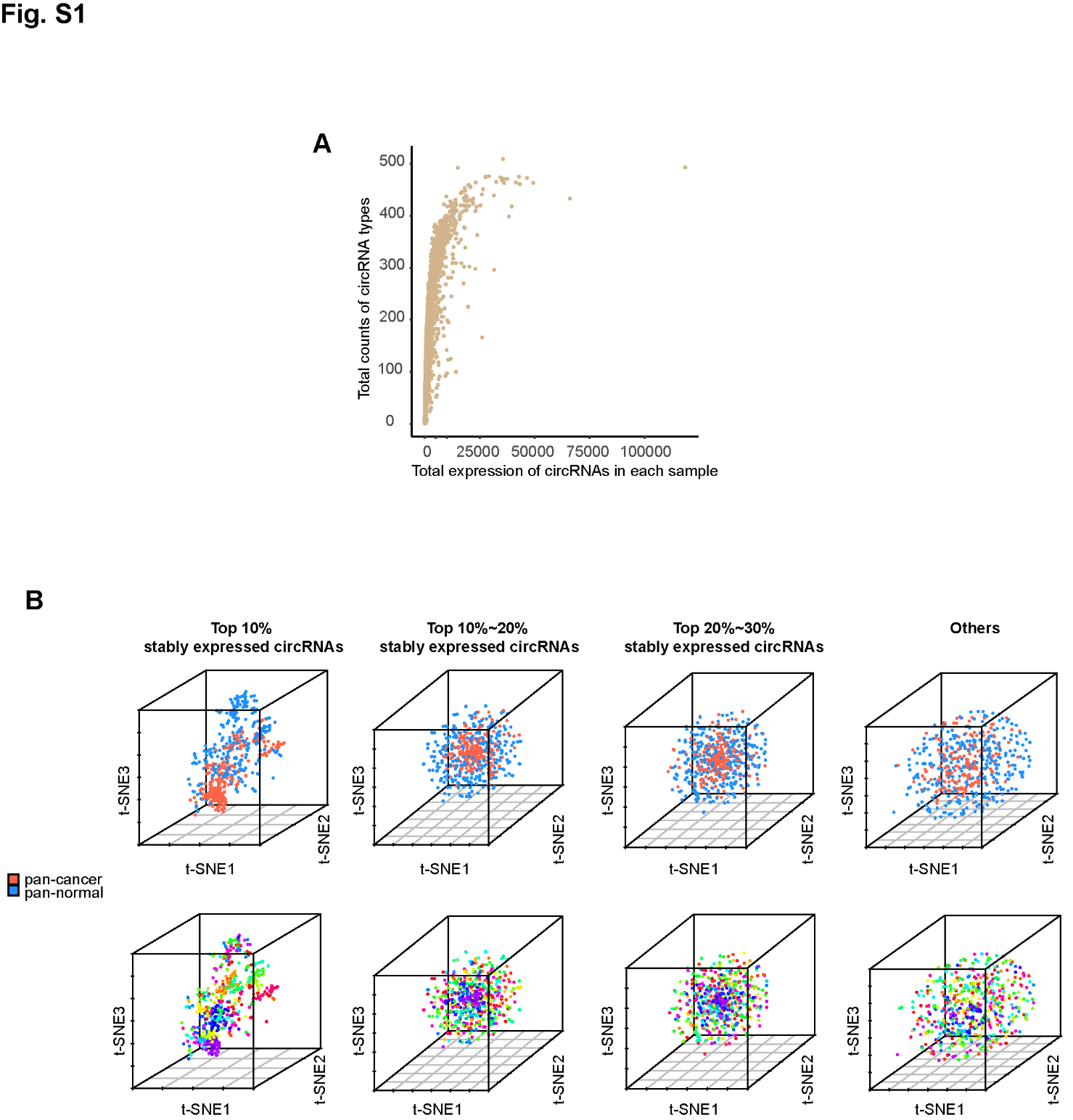


**Figure S1.** Supplementary figure for Figure 1. **a.** Relationship between total circRNA expression and total count of circRNA types. **b**. t-SNE embedding of circRNA profiles in 584 tissue samples, without PCA. The top 10%, top 10%‒20%, top 20%‒30% of the stably expressed circRNAs and others were analyzed separately. Red: cancer tissues; blue: normal tissues. Rainbow: tissue types.


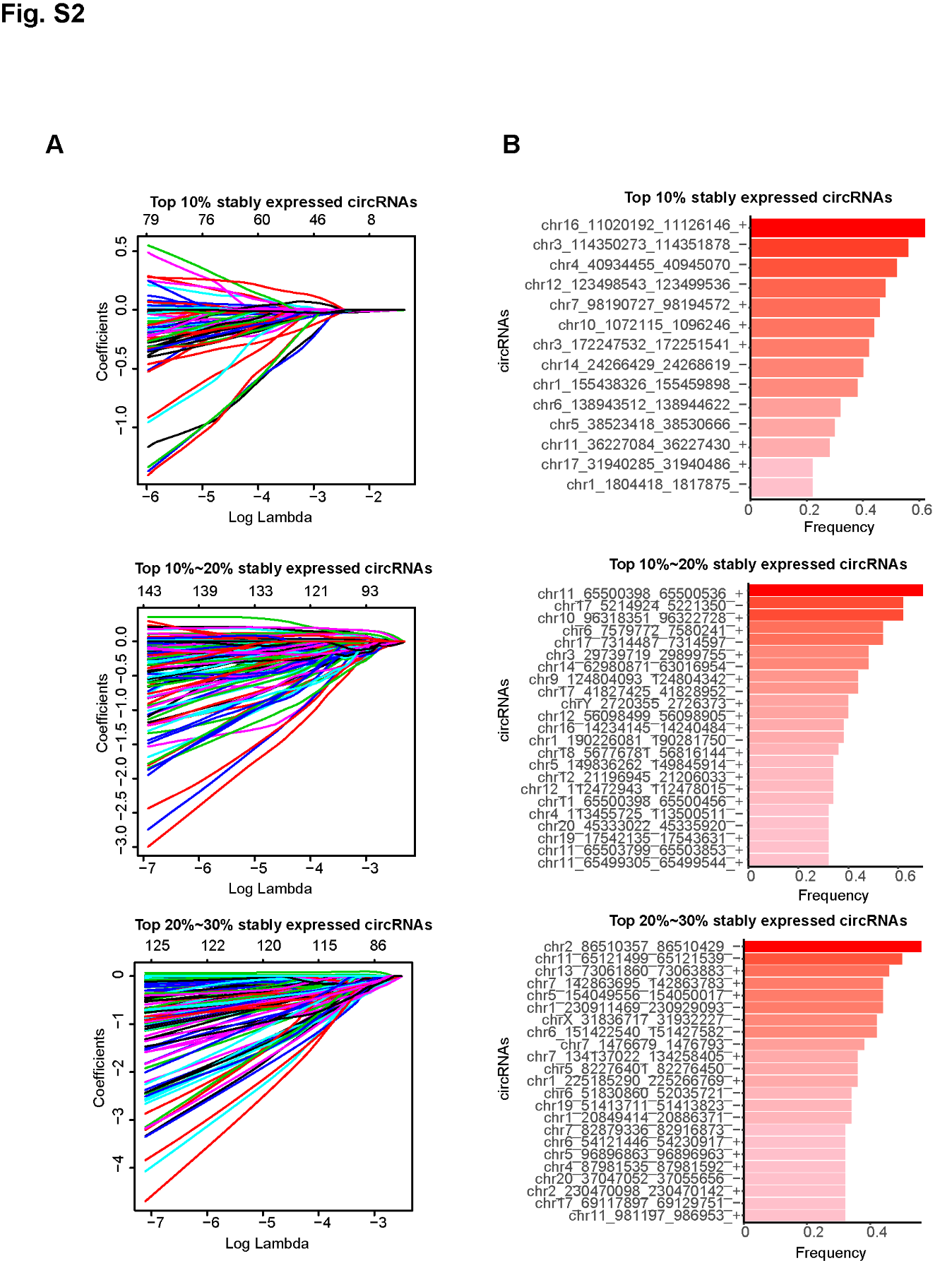


**Figure S2.** Supplementary figure for Figure 3. **a.** Correlation between coefficient and log lambda values in LASSO regression analysis with five-fold cross-validation. The top 10%, top 10%‒20%, and top 20%‒30% of the stably expressed circRNAs were analyzed separately. **b.** Frequency of the top selected circRNAs during the 50 repetitions of LASSO regression. The top 10%, top 10%‒20%, and top 20%‒30% of the stably expressed circRNAs were analyzed separately.


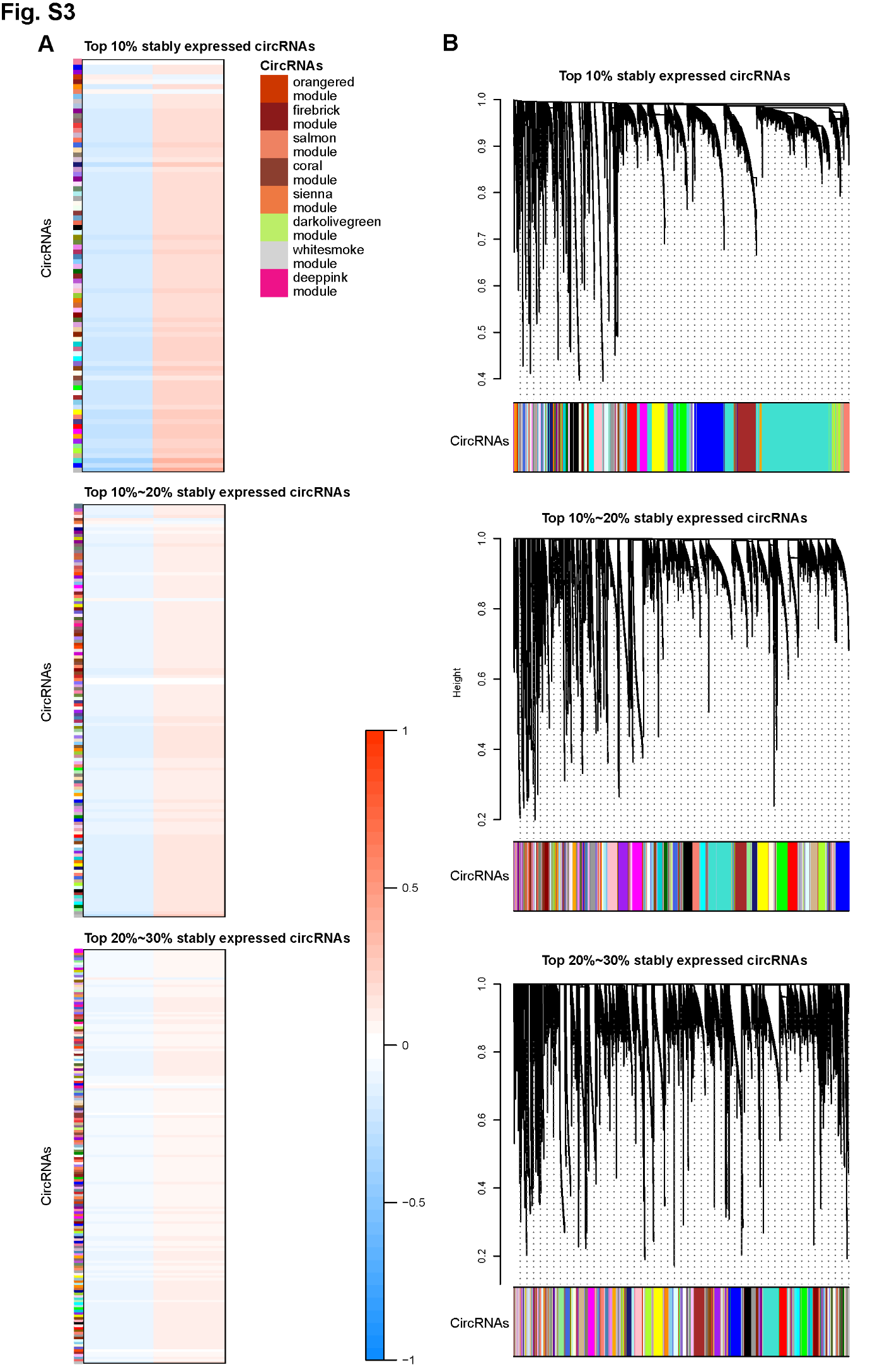


**Figure S3.** Supplementary figure for Figure 4, part A. **a**. Heatmap illustrating the correlation between the circRNA co-expression modules and sample type. The top 10% top 10%‒20%, and top 20%‒30% of the stably expressed circRNAs were analyzed separately. The rainbow-colored bars represent different co-expression modules. **b**. Cluster dendrograms demonstrating the clustering process during the division of circRNA co-expression modules. Rainbow-colored bars represent different co-expression modules.


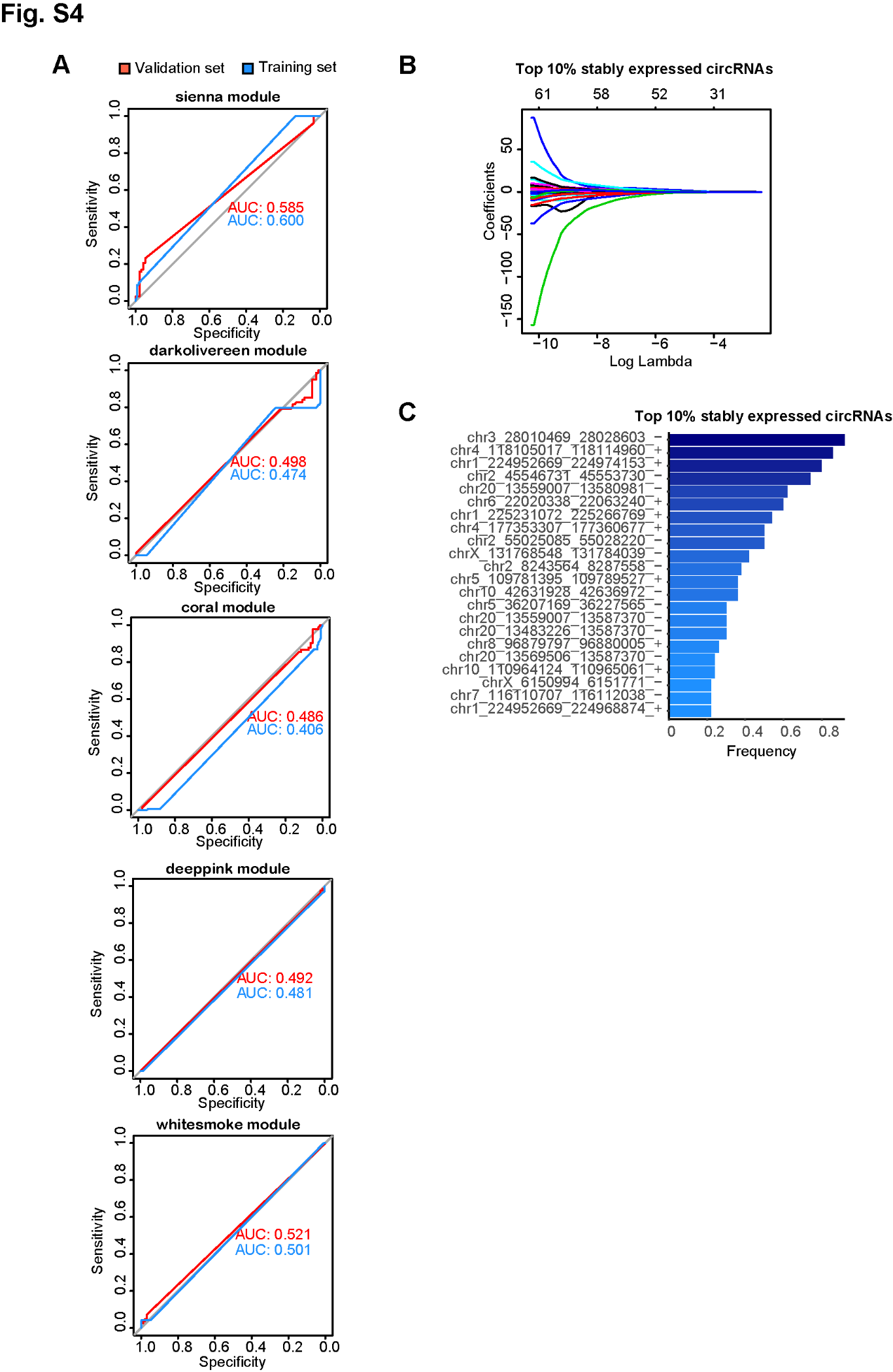


**Figure S4.** Supplementary figure for Figure 4, part B. **a**. Receiver operating characteristic curve showing the ability of cancer tissue-enriched circRNAs to classify cancer and normal tissues via the support vector machine model. Different circRNA co-expression modules were computed separately. Blue: training set; red: validation set. **b.** Correlation between coefficient and log lambda values in the LASSO regression analysis with five-fold cross-validation. The cancer tissue-enriched circRNAs among the top 10% stably expressed circRNAs were further selected. **c.** Frequency of the top selected circRNAs during 50 repetitions of the LASSO regression. Cancer tissue-enriched circRNAs among the top 10% stably expressed circRNAs were further selected.

**Table S1 P**an-cancer tissues and pan-normal tissues provided by the CSCD (IDCSC) database.

Additional File 2 ‘Table S1’

**Table S2** Cancer-specific circRNAs associated with protein-coding genes

Additional File 2 ‘Table S2’

**Table S3** Pan-cancer tissues and pan-normal tissues provided by the MiOncoCirc database.

Additional File 2 ‘Table S3’

**Table S4** Sequencing depth of samples provided by the CSCD (IDCSC) database.

Additional File 2 ‘Table S4’

**Metrics of the Support Vector Machines**

**Figure 3b-1**

> train.con

Confusion Matrix and Statistics

Reference

Prediction cancer normal

cancer 143 17

normal 40 208

Accuracy : 0.8603

95% CI : (0.8228, 0.8924)

No Information Rate : 0.5515

P-Value [Acc > NIR] : < 2.2e-16

Kappa : 0.7142

Mcnemar's Test P-Value : 0.003569

Sensitivity : 0.7814

Specificity : 0.9244

Pos Pred Value : 0.8937

Neg Pred Value : 0.8387

Prevalence : 0.4485

Detection Rate : 0.3505

Detection Prevalence : 0.3922

Balanced Accuracy : 0.8529

'Positive' Class : cancer

> valid.con

Confusion Matrix and Statistics

Reference

Prediction cancer normal

cancer 57 14

normal 25 80

Accuracy : 0.7784

95% CI : (0.7098, 0.8374)

No Information Rate : 0.5341

P-Value [Acc > NIR] : 1.588e-11

Kappa : 0.5509

Mcnemar's Test P-Value : 0.1093

Sensitivity : 0.6951

Specificity : 0.8511

Pos Pred Value : 0.8028

Neg Pred Value : 0.7619

Prevalence : 0.4659

Detection Rate : 0.3239

Detection Prevalence : 0.4034

Balanced Accuracy : 0.7731

'Positive' Class : cancer

**Figure 3b-2**

> train.con

Confusion Matrix and Statistics

Reference

Prediction cancer normal

cancer 180 90

normal 3 135

Accuracy : 0.7721

95% CI : (0.7282, 0.8119)

No Information Rate : 0.5515

P-Value [Acc > NIR] : < 2.2e-16

Kappa : 0.5588

Mcnemar's Test P-Value : < 2.2e-16

Sensitivity : 0.9836

Specificity : 0.6000

Pos Pred Value : 0.6667

Neg Pred Value : 0.9783

Prevalence : 0.4485

Detection Rate : 0.4412

Detection Prevalence : 0.6618

Balanced Accuracy : 0.7918

'Positive' Class : cancer

> valid.con

Confusion Matrix and Statistics

Reference

Prediction cancer normal

cancer 69 51

normal 13 43

Accuracy : 0.6364

95% CI : (0.5606, 0.7074)

No Information Rate : 0.5341

P-Value [Acc > NIR] : 0.003876

Kappa : 0.2903

Mcnemar's Test P-Value : 3.746e-06

Sensitivity : 0.8415

Specificity : 0.4574

Pos Pred Value : 0.5750

Neg Pred Value : 0.7679

Prevalence : 0.4659

Detection Rate : 0.3920

Detection Prevalence : 0.6818

Balanced Accuracy : 0.6495

'Positive' Class : cancer

**Figure 3b-3**

> train.con

Confusion Matrix and Statistics

Reference

Prediction cancer normal

cancer 37 0

normal 146 225

Accuracy : 0.6422

95% CI : (0.5935, 0.6887)

No Information Rate : 0.5515

P-Value [Acc > NIR] : 0.0001241

Kappa : 0.2185

Mcnemar's Test P-Value : < 2.2e-16

Sensitivity : 0.20219

Specificity : 1.00000

Pos Pred Value : 1.00000

Neg Pred Value : 0.60647

Prevalence : 0.44853

Detection Rate : 0.09069

Detection Prevalence : 0.09069

Balanced Accuracy : 0.60109

'Positive' Class : cancer

> valid.con

Confusion Matrix and Statistics

Reference

Prediction cancer normal

cancer 9 5

normal 73 89

Accuracy : 0.5568

95% CI : (0.4802, 0.6315)

No Information Rate : 0.5341

P-Value [Acc > NIR] : 0.2989

Kappa : 0.0597

Mcnemar's Test P-Value : 3.293e-14

Sensitivity : 0.10976

Specificity : 0.94681

Pos Pred Value : 0.64286

Neg Pred Value : 0.54938

Prevalence : 0.46591

Detection Rate : 0.05114

Detection Prevalence : 0.07955

Balanced Accuracy : 0.52828

'Positive' Class : cancer

**Figure 4b-1**

> train.con

Confusion Matrix and Statistics

Reference

Prediction cancer normal

cancer 155 49

normal 28 176

Accuracy : 0.8113

95% CI : (0.7699, 0.8481)

No Information Rate : 0.5515

P-Value [Acc > NIR] : < 2e-16

Kappa : 0.6225

Mcnemar's Test P-Value : 0.02265

Sensitivity : 0.8470

Specificity : 0.7822

Pos Pred Value : 0.7598

Neg Pred Value : 0.8627

Prevalence : 0.4485

Detection Rate : 0.3799

Detection Prevalence : 0.5000

Balanced Accuracy : 0.8146

'Positive' Class : cancer

> valid.con

Confusion Matrix and Statistics

Reference

Prediction cancer normal

cancer 50 40

normal 32 54

Accuracy : 0.5909

95% CI : (0.5144, 0.6643)

No Information Rate : 0.5341

P-Value [Acc > NIR] : 0.0752

Kappa : 0.1831

Mcnemar's Test P-Value : 0.4094

Sensitivity : 0.6098

Specificity : 0.5745

Pos Pred Value : 0.5556

Neg Pred Value : 0.6279

Prevalence : 0.4659

Detection Rate : 0.2841

Detection Prevalence : 0.5114

Balanced Accuracy : 0.5921

'Positive' Class : cancer

**Figure 4b-2**

> train.con

Confusion Matrix and Statistics

Reference

Prediction cancer normal

cancer 109 51

normal 74 174

Accuracy : 0.6936

95% CI : (0.6464, 0.738)

No Information Rate : 0.5515

P-Value [Acc > NIR] : 2.939e-09

Kappa : 0.3733

Mcnemar's Test P-Value : 0.0491

Sensitivity : 0.5956

Specificity : 0.7733

Pos Pred Value : 0.6812

Neg Pred Value : 0.7016

Prevalence : 0.4485

Detection Rate : 0.2672

Detection Prevalence : 0.3922

Balanced Accuracy : 0.6845

'Positive' Class : cancer

> valid.con

Confusion Matrix and Statistics

Reference

Prediction cancer normal

cancer 40 22

normal 42 72

Accuracy : 0.6364

95% CI : (0.5606, 0.7074)

No Information Rate : 0.5341

P-Value [Acc > NIR] : 0.003876

Kappa : 0.2578

Mcnemar's Test P-Value : 0.017549

Sensitivity : 0.4878

Specificity : 0.7660

Pos Pred Value : 0.6452

Neg Pred Value : 0.6316

Prevalence : 0.4659

Detection Rate : 0.2273

Detection Prevalence : 0.3523

Balanced Accuracy : 0.6269

'Positive' Class : cancer

**Figure 4b-3**

> train.con

Confusion Matrix and Statistics

Reference

Prediction cancer normal

cancer 117 70

normal 66 155

Accuracy : 0.6667

95% CI : (0.6186, 0.7123)

No Information Rate : 0.5515

P-Value [Acc > NIR] : 1.399e-06

Kappa : 0.3276

Mcnemar's Test P-Value : 0.797

Sensitivity : 0.6393

Specificity : 0.6889

Pos Pred Value : 0.6257

Neg Pred Value : 0.7014

Prevalence : 0.4485

Detection Rate : 0.2868

Detection Prevalence : 0.4583

Balanced Accuracy : 0.6641

'Positive' Class : cancer

> valid.con

Confusion Matrix and Statistics

Reference

Prediction cancer normal

cancer 45 48

normal 37 46

Accuracy : 0.517

95% CI : (0.4406, 0.5929)

No Information Rate : 0.5341

P-Value [Acc > NIR] : 0.7019

Kappa : 0.0378

Mcnemar's Test P-Value : 0.2781

Sensitivity : 0.5488

Specificity : 0.4894

Pos Pred Value : 0.4839

Neg Pred Value : 0.5542

Prevalence : 0.4659

Detection Rate : 0.2557

Detection Prevalence : 0.5284

Balanced Accuracy : 0.5191

'Positive' Class : cancer

**Figure 4c**

> train.con

Confusion Matrix and Statistics

Reference

Prediction cancer normal

cancer 165 41

normal 18 184

Accuracy : 0.8554

95% CI : (0.8175, 0.8881)

No Information Rate : 0.5515

P-Value [Acc > NIR] : < 2.2e-16

Kappa : 0.7111

Mcnemar's Test P-Value : 0.004181

Sensitivity : 0.9016

Specificity : 0.8178

Pos Pred Value : 0.8010

Neg Pred Value : 0.9109

Prevalence : 0.4485

Detection Rate : 0.4044

Detection Prevalence : 0.5049

Balanced Accuracy : 0.8597

'Positive' Class : cancer

> valid.con

Confusion Matrix and Statistics

Reference

Prediction cancer normal

cancer 47 28

normal 35 66

Accuracy : 0.642

95% CI : (0.5665, 0.7128)

No Information Rate : 0.5341

P-Value [Acc > NIR] : 0.002425

Kappa : 0.2768

Mcnemar's Test P-Value : 0.449692

Sensitivity : 0.5732

Specificity : 0.7021

Pos Pred Value : 0.6267

Neg Pred Value : 0.6535

Prevalence : 0.4659

Detection Rate : 0.2670

Detection Prevalence : 0.4261

Balanced Accuracy : 0.6376

'Positive' Class : cancer

**Session Info of R software**

R version 3.6.0 (2019-04-26)

Platform: x86_64-w64-mingw32/x64 (64-bit)

Running under: Windows >= 8 x64 (build 9200)

Matrix products: default

locale:

[1] LC_COLLATE=Chinese (Simplified)_China.936 LC_CTYPE=Chinese (Simplified)_China.936

[3] LC_MONETARY=Chinese (Simplified)_China.936 LC_NUMERIC=C

[5] LC_TIME=Chinese (Simplified)_China.936

attached base packages:

[1] stats4 parallel grid stats graphics grDevices utils datasets methods base

other attached packages:

[1] Rmisc_1.5 plyr_1.8.6 psych_2.0.9 ggExtra_0.9

[5] devtools_2.3.2 usethis_1.6.3 ggfortify_0.4.11 DESeq2_1.24.0

[9] SummarizedExperiment_1.14.1 DelayedArray_0.10.0 BiocParallel_1.18.1 matrixStats_0.57.0

[13] Biobase_2.44.0 GenomicRanges_1.36.1 GenomeInfoDb_1.20.0 IRanges_2.18.3

[17] S4Vectors_0.22.1 BiocGenerics_0.30.0 tidyr_1.1.2 dplyr_1.0.2

[21] ggpubr_0.4.0 RColorBrewer_1.1-2 stringr_1.4.0 reshape2_1.4.4

[25] WGCNA_1.69 fastcluster_1.1.25 dynamicTreeCut_1.63-1 pheatmap_1.0.12

[29] e1071_1.7-3 caret_6.0-86 lattice_0.20-41 varSelRF_0.7-8

[33] randomForest_4.6-14 pROC_1.16.2 glmnet_4.0-2 Matrix_1.2-17

[37] scatterplot3d_0.3-41 Rtsne_0.15 VennDiagram_1.6.20 futile.logger_1.4.3

[41] ggplot2_3.3.2

loaded via a namespace (and not attached):

[1] tidyselect_1.1.0 RSQLite_2.2.1 AnnotationDbi_1.46.1 htmlwidgets_1.5.2 munsell_0.5.0

[6] codetools_0.2-16 preprocessCore_1.46.0 miniUI_0.1.1.1 withr_2.3.0 colorspace_1.4-1

[11] knitr_1.30 rstudioapi_0.11 ggsignif_0.6.0 GenomeInfoDbData_1.2.1 mnormt_2.0.2

[16] bit64_4.0.5 rprojroot_1.3-2 vctrs_0.3.4 generics_0.1.0 lambda.r_1.2.4

[21] ipred_0.9-9 xfun_0.18 R6_2.4.1 doParallel_1.0.15 locfit_1.5-9.4

[26] bitops_1.0-6 assertthat_0.2.1 promises_1.1.1 scales_1.1.1 nnet_7.3-12

[31] gtable_0.3.0 processx_3.4.4 timeDate_3043.102 rlang_0.4.10 genefilter_1.66.0

[36] splines_3.6.0 rstatix_0.6.0 ModelMetrics_1.2.2.2 impute_1.58.0 broom_0.7.1

[41] checkmate_2.0.0 yaml_2.2.1 abind_1.4-5 backports_1.1.10 httpuv_1.5.4

[46] Hmisc_4.4-1 tools_3.6.0 lava_1.6.8.1 ellipsis_0.3.1 sessioninfo_1.1.1

[51] Rcpp_1.0.5 base64enc_0.1-3 zlibbioc_1.30.0 purrr_0.3.4 RCurl_1.98-1.2

[56] ps_1.3.4 prettyunits_1.1.1 rpart_4.1-15 haven_2.3.1 cluster_2.0.8

[61] fs_1.5.0 magrittr_1.5 data.table_1.13.0 futile.options_1.0.1 openxlsx_4.2.2

[66] tmvnsim_1.0-2 pkgload_1.1.0 hms_0.5.3 mime_0.9 xtable_1.8-4

[71] XML_3.99-0.3 rio_0.5.16 jpeg_0.1-8.1 readxl_1.3.1 gridExtra_2.3

[76] shape_1.4.5 testthat_3.0.2 compiler_3.6.0 tibble_3.0.3 crayon_1.3.4

[81] htmltools_0.5.0 later_1.1.0.1 Formula_1.2-3 geneplotter_1.62.0 lubridate_1.7.9

[86] DBI_1.1.0 formatR_1.7 MASS_7.3-51.4 car_3.0-10 cli_2.3.0

[91] gower_0.2.2 forcats_0.5.0 pkgconfig_2.0.3 foreign_0.8-71 recipes_0.1.15

[96] foreach_1.5.0 annotate_1.62.0 XVector_0.24.0 prodlim_2019.11.13 callr_3.5.1

[101] digest_0.6.25 cellranger_1.1.0 htmlTable_2.1.0 curl_4.3 shiny_1.5.0

[106] lifecycle_0.2.0 nlme_3.1-139 carData_3.0-4 desc_1.2.0 pillar_1.4.6

[111] fastmap_1.0.1 pkgbuild_1.1.0 survival_3.2-7 GO.db_3.8.2 glue_1.4.2

[116] remotes_2.2.0 zip_2.1.1 png_0.1-7 iterators_1.0.12 bit_4.0.4

[121] class_7.3-15 stringi_1.4.6 blob_1.2.1 latticeExtra_0.6-29 memoise_1.1.0
